# Dr. William Lomax

**Published:** 1890-09

**Authors:** 


DR. WILLIAM LOMAX, of Marion, Ind., has recently endowed the Indiana Medical College with the generous sum of $100,000, encumbering the gift only to the extent of an annuity of $1,200 to himself and wife as long as either shall live. This example is worthy of imitation in other cities where medical colleges are in urgent need of substantial encouragement in their work of educating young men for the profession of medicine.

